# The Treatment of Heart Disease

**Published:** 1895-08-24

**Authors:** Theodore Fisher


### The Treatment of Heart Disease.

By Theodore Fisher, M.D.Lond.

The general character of the methods practised by Dr. Schott at Nauheim is now so well known that careful description is scarcely necessary, yet to some a brief outline may be of value in view of the attention which has been attracted to the subject recently. The system comprises bathing in a mineral water, associated with slowly-conducted exercises. The water, which is strongly impregnated with common salt, is also charged with large quantities of carbonic acid gas, the presence of which causes the water to escape from a depth of several hundred feet below the earth's surface at considerable pressure. The water is conveyed to the baths by this natural pressure, and when the tap allowing its entrance into a bath is turned on, the foaming water rushes in with such rapidity and force that, unless care is exercised, spray may be scattered over the bath-room. After the tap has been turned off the turmoil quickly ceases, but multitudes of small bubbles rising through the clear water reveal the presence of carbonic acid gas.

Baths of this nature are not, however, often given to patients on their arrival at Nauheim. Water from which the carbonic acid gas has been allowed to escape is first used. Such water contains about 1 per cent. of sodium chloride, but this salt is added in later baths until the strength reaches from 2 to 3 per cent. After a varying number of such saline baths the patient will probably be ordered those containing carbonic acid gas. These are of two kinds. In one, the tap having been turned off, the amount of carbonic acid grows gradually less during the period of immersion; in the other —the effervescing running baths—continual ingress of the gas-laden water is allowed. A not unimportant feature of the baths is their temperature. The natural temperature of the two springs which supply the baths is from 82 to 95 deg. Fah. Baths of between 92 and 95 deg. Fah. are most commonly used. Associated with the baths are exercises, designed by Dr. Schott, with the object of bringing nearly every muscle in the body into play. The movements are performed slowly, and slight resistance is offered by an attendant. The patient should keep his head erect, and holding of the breath is to be carefully avoided.

Bathing may seem strange treatment for diseases of the heart, but a visit to Nauheim, and conversation with the patients there, will convince the inquirer that real improvement of marked character takes place. Thus one gentleman told me that when he first went to Nanheim he could not walk a few yards without having to stop on account of shortness of breath, yet on the first afternoon of my visit he kindly offered to show me round Nauheim, when it was found that he could walk up-hill almost as well as I could myself. Another gentleman on arrival at Nauheim was unable to walk from his hotel to the bath-house without a rest on the way, but since his first visit he had walked fourteen miles. Perhaps the opinion of a third, who took a somewhat pessimistic view of his own case, is worthy of mention. He could not doubt the efficacy of the treatment, and as an instance of its value mentioned the case of his baker, who two or three years before had been thought to be dying. Œdema was said to have been present to an extreme degree, but although ordinary methods of treatment had failed to give relief, at Nauheim he recovered strength, and at the time the information was given was "walking about almost like an ordinary man."

A question may suggest itself as to the nature of the heart affections that derive benefit. Dr. Schott considers that nearly every kind of heart disease is materially improved. The writer is disposed to think with Dr. Wethered, who paid a visit to Nauheim, and read a paper upon its treatment at the annual meeting of the British Medical Association in 1894, that cases of dilatation of the heart unassociated with valvular disease improve most markedly. In connection with this point of enlargement of the heart without valvular disease, it may be mentioned that the writer has examined the post-mortem reports of nearly all cases of death from heart disease in Guy's Hospital covering a period of twenty years. Above the age of forty any deformity of the mitral valves was a comparatively rare lesion. If we are able to draw general conclusions from those records, we may say that in the great majority of cases of mitral regurgitation occurring in patients over forty valvular disease is absent. The wide and lengthened experience of Dr. Schott enables him to say, however, that affections of the heart in which valvular lesions are present are greatly benefited by the treatment.

While at present the treatment by baths and exercises is almost limited to Nauheim, Dr. Schott considers that artificially prepared baths will prove useful. He takes a pleasure in showing doctors the methods of treatment, in order that they may be led to adopt them. Dr. Babcock, of Chicago, who himself paid a visit to Nauheim as a patient, has treated cases with satisfactory results, and Dr. Wethered has treated others under the care of Dr. Douglas Powell and Dr. Cayley at the Middlesex Hospital. Provision will no doubt be made in various places in England for the carrying out of the treatment. We believe that in Harrogate this has already been done, and that in the new Spa Baths, now in course of erection at Clifton, arrangements are contemplated for the application of the treatment. In private practice, however, it will probably be found difficult to carry it out efficiently. But it may be added that those who feel disposed to test the value of the system will find the method of preparation of artificial baths described in the book of Dr. Bezly Thorne. In the same book are carefully drawn illustrations of the exercises that are generally associated with the treatment by baths.

